# Vaccination Experiences

**Published:** 1881-09

**Authors:** 


### VACCINATION EXPERIENCES.

A Detroit (Mich.) doctor, who has vaccinated over five hundred persons, reports that not more than two men out of ten have it done in a straightforward way. They hesitate, make inquiries, and postpone it a few days. One insisted upon being strapped fast to a chair while the operation was performed. Another wanted to take chloroform. A negro was one day seen to walk past the office several times, and the doctor finally stepped to the door and asked him if he wanted to be vaccinated. "'Deed, sah, dat's what I cum for," he replied, "but de werry minit I turned dat corner de blamed thing quit aching!" As a rule, the men, when they feel the lancet, cry "Woosh!" or "Thunder?" The women cry "Ouch!" generally, but now and then one screams "Oh, Lordy!"

Enforced vaccination in England has led almost to a riot in the coal and iron district near Manchester. The report is now current that the Government find the population of the country is increasing too rapidly, and are determined to destroy as many as possible of the rising generation. Ready credence is given to other stories of the most monstrous character, and the people, in fact, are reduced to that state of panic in which they will lend an ear to any story, however absurd.

